# Histone Deacetylase Inhibitor Panobinostat Benefits the Therapeutic Efficacy of Oncolytic Herpes Simplex Virus Combined with PD-1/PD-L1 Blocking in Glioma and Squamous Cell Carcinoma Models

**DOI:** 10.3390/v14122796

**Published:** 2022-12-15

**Authors:** Yinglin Wu, Xiaoqing Chen, Lei Wang, Xusha Zhou, Yonghong Liu, Dongmei Ji, Peigen Ren, Grace Guoying Zhou, Jing Zhao

**Affiliations:** 1 Department of Immunology, School of Basic Medical Sciences, Guangzhou Medical University, Guangzhou 511436, China; 2 Shenzhen International Institute for Biomedical Research, Shenzhen 518110, China; 3 Research Center for Reproduction and Health Development, Institute of Biomedicine and Biotechnology, Shenzhen Institutes of Advanced Technology, Chinese Academy of Sciences, Shenzhen 518055, China; 4 Department of Medical Oncology, Shanghai Cancer Center and Shanghai Medical College, Fudan University, Shanghai 200032, China

**Keywords:** oHSV, HDACi panobinostat, glioma, squamous cell carcinoma

## Abstract

Background: Combination therapy has been widely explored for oncolytic virus (OV), as it can be met with tumor resistance. The HDAC inhibitor (HDACi) panobinostat is a potent pan-deacetylase inhibitor which blocks multiple cancer-related pathways and reverses epigenetic events in cancer progression. Methods: In this study, oncolytic activity in vitro and antitumor therapeutic efficacy in vivo when combined with oHSV and panobinostat were investigated. Results: (1) Treatment with panobinostat enhanced oHSV propagation and cytotoxicity in human glioma A172 and squamous cell carcinoma SCC9 cells. (2) Combined treatment with oHSV and panobinostat enhanced virus replication mediated by the transcriptional downregulation of IFN-β- and IFN-responsive antiviral genes in human glioma A172 and squamous cell carcinoma SCC9 cells. (3) Panobinostat treatment induced upregulation of PD-L1 expression in both glioma and squamous cell carcinoma cells. (4) A significantly enhanced therapeutic efficacy was shown in vivo for the murine glioma CT-2A and squamous cell carcinoma SCC7 models when treated with a combination of oHSV, including PD-1/PD-L1 blockade and HDAC inhibition. Conclusions: Consequently, these data provide some new clues for the clinical development of combination therapy with OVs, epigenetic modifiers, and checkpoint blockades for glioma and squamous cell carcinoma.

## 1. Introduction

Oncolytic virus (OV) with naturally inherited or engineered properties is emerging as a therapeutic agent in oncology [1,2]. Oncolytic herpes simplex virus (oHSV), as a member of OVs, can lyse tumor cells directly with no damage to healthy cells. What is more, oHSV can be genetically engineered to arm with some payloads, e.g., cytokines, chemokines, immune checkpoints inhibitors, and Chimeric antigen receptor (CAR) antigens [3]. However, the resistance of tumor cells to oHSV oncolysis is still a crucial barrier to the antitumor response, which may be due to a lower viral susceptibility, innate antiviral immune responses, and attenuated apoptotic tumor death [4,5,6]. Thus, the development of combination therapy with therapeutic modalities to address these resistance mechanisms to OVs and to enhance oncolytic virotherapy is becoming a widely explored therapy choice for antitumor research and clinical trials [7,8].

Emerging evidence shows that tumors commonly hijack various epigenetic mechanisms to escape immune restriction [9], and epigenetic inhibitors have been validated for single use or in combination with other drugs in oncologic therapeutics [10,11]. There are four major mechanisms of epigenetic regulation: DNA methylation, histone posttranslational modifications, chromatin structure regulation, and noncoding RNA regulation [9]. Histone deacetylase (HDAC) enzymes involving histone posttranslational modifications play a critical role in the epigenetic regulation of cellular functions and signaling pathways in cancers [10]. Thus, HDAC inhibitors (HDACi) have emerged as a promising new class of multifunctional anticancer agents via repressive changes in chromatin structure and deacetylation of various host transcription factors, as well as immunomodulation of tumor microenvironment [12,13,14].

Pre-clinical studies have shown that HDAC inhibition can induce enhancement of oncolytic virus infection in many types of cancers, including glioma and squamous cell carcinoma models [15,16]. It has been reported that the HDACi Scriptaid and panobinostat (LBH589) combined with the oncolytic virus Delta24-RGD exert enhanced anti-tumor efficacy in patient-derived glioblastoma cells [17]. Akihiro Otsuki et al. reported that HDACi VPA pretreatment augments the propagation of oHSV and improves the therapeutic efficacy of oHSV in a human glioma xenograft model in vivo [18]. In a head-and-neck squamous cell carcinoma model, HDACi SAHA treatment increased reovirus entry and enhanced anti-tumor immune responses [19]. In an oral squamous cell carcinoma model, HDACi TSA has been shown to be an enhancing agent for oncolytic virotherapy with γ34.5 gene-deficient HSV-1 [20]. In addition, the clinical combination of talimogene laherparepvec (HSV-encoding GM-CSF) with VPA has shown enhanced oncolysis and antitumor immunity in melanoma [16]. The HDACi VPA mentioned above is a more class I-specific HDACi, while TSA is a class I, IIa, and IIb HDACi.

Another HDACi, panobinostat used in this study, is a potent pan-deacetylase inhibitor with activity against class I, II, and IV HDAC enzymes, and was approved in 2015 by FDA for the treatment of multiple myeloma [21]. It has been reported that panobinostat can block multiple cancer-related pathways and reverse the epigenetic events implicated in cancer progression [22]. Furthermore, it has been reported that LBH589 can induce the upregulation of PD-L1 and PD-L2 in melanoma cells in vitro and in a B16F10 mouse model in vivo [23]. Here, in this study, the HDACi panobinostat was used in combination with oHSV T3855, an oHSV expressing mouse IL-12, and an anti-mouse PD-1 antibody [24], and both the oncolytic virus replication in vitro and the oncolytic activity in vivo in glioma and squamous cell carcinoma were investigated.

## 2. Materials and Methods

### 2.1. Cell Lines and Virus

HEp-2 and SCC7 cells were obtained from the University of Chicago. HEp-2 cells were routinely cultured in DMEM (Cat. C11995500BT, Life Technologies, Grand Island, NY, USA) supplemented with 5% (vol/vol) Fetal Bovine Serum (FBS, Cat.10270-106, Gibco, Grand Island, NY, USA) and SCC7 was maintained in RPMI-1640 (Cat. C11875500BT, Life Technologies, Grand Island, NY, USA) containing 10% (vol/vol) FBS. A172, D54, and Tca8113 cells were purchased from JOINN Biologics, SCC9 cells were purchased from the BeNa Culture Collection, and CT2A cells were purchased from BLUEFBIO. A172, D54, SCC9, and CT2A cells were routinely cultured in DMEM supplemented with 10% (vol/vol) FBS. Tca8113 cells were maintained in RPMI-1640 containing 10% (vol/vol) FBS. Vero cells were purchased from the American Type Culture Collection (ATCC, Cat. CCL-81, Manassas, VA, USA) and cultured in DMEM supplemented with 5% newborn calf serum (NBCS, Cat. NCD500, ExCell Bio, Shanghai, China). All culture media were supplemented with 100 U/mL of penicillin and 100 μg/mL of streptomycin.

The oncolytic herpes viruses (oHSV) T1012G and T3855 were constructed and reported elsewhere [24]. In detail, T3855 was constructed to encode murine IL-12 and anti-PD-1 antibody, based on the backbone of T1012G.

### 2.2. Antibodies

The antibodies used in this study included acetyl-histone H3 (Lys9) (Cat. 9649T, Cell Signaling Technology, Danvers, MA, USA), acetyl-histone H4 (Lys8) (Cat. 594T, Cell Signaling Technology, Danvers, MA, USA), anti-PD-L1 (Cat. Ab238697, abcam, Cambridge, MA, USA), anti-GAPDH (Cat. 2118S, Cell Signaling Technology, Danvers, MA, USA), anti-β-actin (Cat. 20536-1-AP, Proteintech, Rosemont, IL, USA), and anti-nectin-1 (Cat. 37-5900, Invitrogen, Waltham, MA, USA).

### 2.3. Cell Counting Kit 8 assay

Tumor-cell-killing activity was assessed using a CCK8 assay. Briefly, A172, D54, SCC9, or Tca8113 cells were cultured in 96-well plates at 5 × 10^3^ cells/well. The cells were pretreated with panobinostat at the final concentrations of 1, 10, and 100 nM for 14 h after the cells had adhered overnight and then infected with T1012G for another 48 h. The cell viability was evaluated using a Cell Counting Kit 8 (Cat. HY-K030, MCE, Monmouth Junction, NJ, USA) according to the manufacturer’s instructions. The cell viability rate was calculated according to the following formula:

$$\mathrm{Cell}\;\mathrm{viability}\;\left(100\%\right)=\frac{A\;\mathrm{value}\;\mathrm{of}\;\mathrm{the}\;\mathrm{experimental}\;\mathrm{wells}\;-\;A\;\mathrm{value}\;\mathrm{of}\;\mathrm{the}\;\mathrm{blank}\;\mathrm{wells}}{A\;\mathrm{value}\;\mathrm{of}\;\mathrm{the}\;\mathrm{target}\;\mathrm{cell}\;\mathrm{wells}\;-\;A\;\mathrm{value}\;\mathrm{of}\;\mathrm{the}\;\mathrm{blank}\;\mathrm{wells}\;}\times 100\%$$

The assay was performed in triplicate.

### 2.4. RNA Extraction and Real-Time PCR

The total RNAs from cells were isolated using TRIzol reagent (Cat. 15596026, ThermoFisher, Waltham, MA, USA), followed by DNase I (Cat. 2270A, Takara, Maebashi, Gunma, Japan) treatment at 37 °C by 10 min and inactivation at 80 °C by 10 min. A total amount of 0.5 µg of RNA was reverse-transcribed to cDNA with oligo(dT) using a PCR RT Kit (Cat. FSQ-101, TOYOBO, Kita-ku, Osaka, Japan) according to the manufacturer’s instructions. The expression of mRNA was determined by real-time PCR using SYBR Premix purchased from TaKaRa. The variability in the expression levels was normalized by 18S rRNA and analyzed using the 2^-ΔΔCT^ method. The primers used in the PCRs were as follows (Table 1).

### 2.5. Immunoblotting Assays

Cells were collected at the indicated times after infection. The procedures for cell harvesting, solubilization, protein quantification, SDS/PAGE, and transfer to polyvinylidene difluoride membranes (Cat.ISEQ00010, Millipore, Bedford, MA, USA) were as described previously [24]. The proteins were detected by incubation with the appropriate primary antibody, followed by horseradish peroxidase (HRP)-conjugated secondary antibody (Cat.31460 or Cat.31430, Invitrogen, Waltham, MA, USA) and the ECL reagent (Cat.WBKLS0500, Merck, Darmstadt, Hessen, Germany). Images were captured using a ChemiDoc Touch Imaging System (Bio-Rad) and processed using ImageLab software.

### 2.6. Virus Titration

A total of 1 × 10^6^ cells (A172, D54, SCC9, Tca8113) were seeded into six-well plates. After 24 h, the adherent cells were pretreated with DMSO or HDACi panobinostat for 14 h and then exposed to 0.1 or 1 PFU/mL of T1012G per cell. The cells were harvested at 48 h after infection. Viral progeny was titrated on Vero cells after three freeze–thaw cycles. In detail, the viral progeny was diluted in medium 199 (Cat.C11150500BT, Gibco, Billings, MT, USA) to the appropriate titer, and then the Vero cells were infected with diluted viral progeny in a 25 cm^2^ flask. Two hours later, change medium with full medium. After 72 h, the viral plaque is counted with confirmation under the microscope, and the viral titer can be obtained. The titers of the viral stock used in this study, T1012G and T3855, are 6 × 10^8^ PFU/mL, 1.8 × 10^8^ PFU/mL, respectively.

### 2.7. Syngeneic Mouse Model

All animal experiments were performed under protocols approved by the Institutional Animal Care and Use Committee of SAFE (Shen Zhen, China) New Drug Research Technology Company. Five-week-old female C3H/HeN and C57BL/6 mice were purchased from Charles River Laboratories (Beijing, China) and housed under specific pathogen-free (SPF) conditions. The syngeneic mice models were C3H/HeN mice for SCC7 and C57BL/6 mice for CT-2A tumors. The SCC7 and CT-2A tumor models were generated by implantation of 2 × 10^6^ cells subcutaneously into the mouse flanks, respectively. Once the mean tumor volumes reached about 80–100 mm^3^, C3H/HeN or C57BL/6 mice (*n* = 8 per group) were randomly grouped and treated with oHSV T3855 (1 *×* 10^6^ or 1 *×* 10^7^ PFU/mouse) or the vehicle control via intratumoral injection on day 1; HDACi panobinostat (5.0 mg/kg) or vehicle control was administered via intraperitoneal injection with a five-day-on and two-day-off regimen for two cycles. The tumor growth was measured in two dimensions, recording the greatest length and width using digital calipers and the tumor volume is equal to length × (width)^2^ × 1/2. The tumor sizes were plotted as the average size for each group.

## 3. Results

### 3.1. HDACi Panobinostat Treatment Enhances the oHSV Propagation and Cytotoxicity in Human Glioma A172 and Squamous Cell Carcinoma SCC9 Cells

The combined use of epigenetic inhibitors with oncolytic virotherapy is a useful way to increase the therapeutic efficiency in several types of cancer [1,2,4]. To evaluate the potential benefit of combining the HDACi panobinostat and oHSV, the oHSV-resistant tumor cell lines, human glioma A172 and D54 cells, and squamous cell carcinoma SCC9 and Tca8113 cells (Figure S1A) were treated with panobinostat at different concentrations. Panobinostat is a potent inhibitor with activity against class I, II, and IV HDAC enzymes, suggesting a pan-HDAC activity [21]. Inducing histone acetylation by inhibition of HDAC activity with panobinostat was verified in A172, D54, SCC9, and Tca8113 cells (Figure S1B).

**Figure 1:**
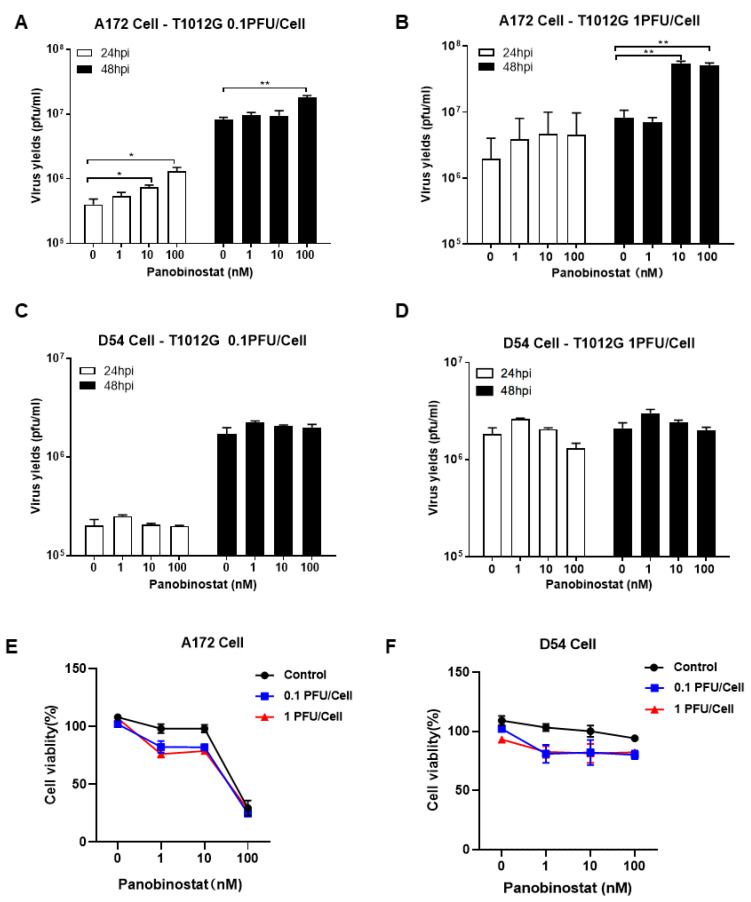
The HDACi panobinostat promoted oHSV replication and tumor-cell killing in human glioma A172 cells. Human glioma cells A172 (**A**,**B**) and D54 (**C**,**D**) were pretreated with panobinostat at concentrations of 1, 10, and 100 nM for 14 h and then infected with T1012G (0.1 or 1 PFU/Cell) for another 48 h, respectively. The infected cell pellets were harvested at 48 hpi. The titration was measured by conventional plaque assay on Vero cells after three freeze–thaw cycles. The tumor-cell-killing activity was assessed using a CCK8 assay. A172 (**E**) and D54 (**F**) cells were mock- or pretreated with panobinostat at different concentrations as above for 14 h and then infected with T1012G at an indicated MOI for 48 h. The cell viability of the infected cells was measured with a CCK8 assay (mean ± SD). The assay was performed in triplicate. Differences between datasets were assessed by Student’s *t*-test (two-tailed) using GraphPad Prism software. * *p* < 0.05 and ** *p* < 0.01.

Notably, the yield of oHSV progeny increased significantly (Figure 1A,B), over six-fold for human glioma cell A172 when pretreated with panobinostat at a concentration of 10 or 100 nM for 14 h and then infected with 1 MOI of T1012G, the backbone of oHSV without payload genes, for another 48 h. For human glioma D54 cells, there was no increase at 0.1 MOI, but a tendency to decrease in oHSV yields at 24 h post-infection (hpi) with 1 MOI when pretreated with panobinostat at 100 nM (Figure 1C,D). In contrast, co-treatment with panobinostat and oHSV showed almost no increase in the viral titer in both A172 and D54 cells (Figure S2). Compared to the mock treatment, the oHSV-infected A172 cells exhibited increased cytotoxicity when pretreated with panobinostat at a concentration of 1 or 10 nM, whereas treatment with 100 nM panobinostat showed a maximum of 75% cytotoxicity whether combined with or without oHSV infection (Figure 1E). For D54 cells, the cell survival rate displayed a trend to decrease when pretreated with panobinostat in a dose-independent manner (Figure 1F). This indicates that enhanced cytotoxicity occurs when pretreated with panobinostat in human glioma cells and shows a diverse sensitivity to glioma cells.

**Figure 2:**
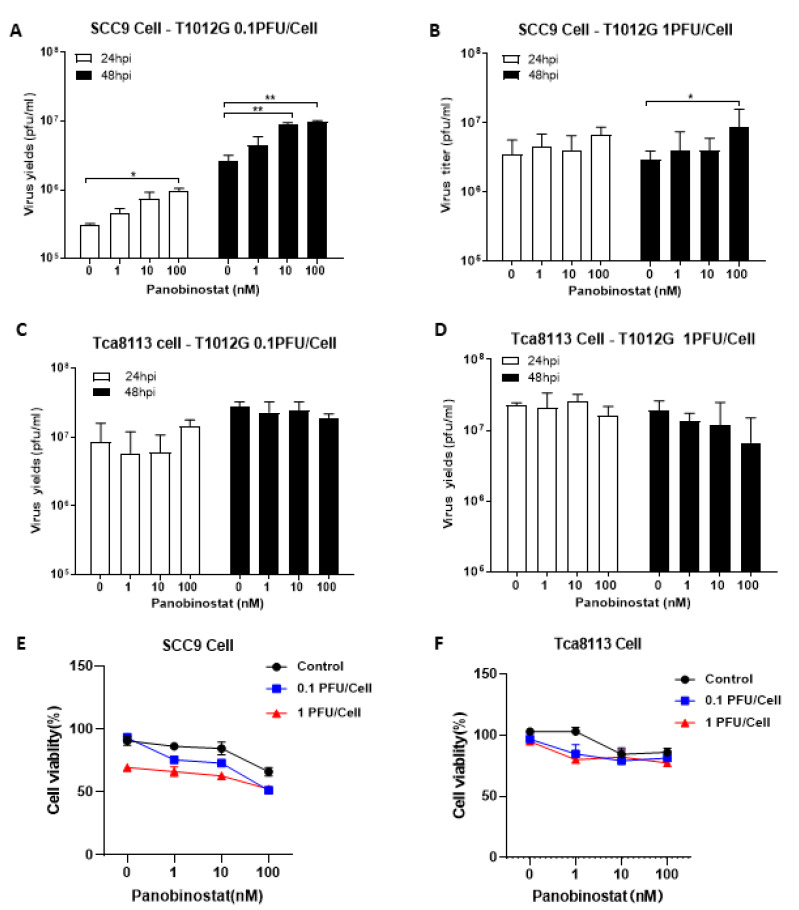
Panobinostat treatment facilitated oHSV replication and tumor cytotoxicity in human squamous cell carcinoma SCC9 cells. Human squamous cell carcinoma SCC9 (**A**,**B**) and Tca8113 cells (**C**,**D**) were pretreated with panobinostat (1, 10, or 100 nM) for 14 h and then infected with T1012G (0.1 or 1 PFU/Cell) for another 48 h, respectively. The infected cell pellets were harvested at 48 hpi. The titration was measured by conventional plaque assay on Vero cells after three freeze–thaw cycles. The cytotoxicity activity was assessed using a CCK8 assay. SCC9 (**E**) and Tca8113 (**F**) cells were mock- or pretreated with panobinostat as above, following infection with T1012G for 48 h. The cell viability of the infected cells was measured with a CCK8 assay (mean ± SD). The assay was performed in triplicate. Differences between datasets were assessed by Student’s *t*-test (two-tailed) using GraphPad Prism software. * *p* < 0.05 and ** *p* < 0.01.

When pretreated with panobinostat in squamous cell carcinoma SCC9 cells, the oHSV replication displayed a similar pattern to the A172 cells, showing a significant increase in the viral yield (Figure 2A,B). In contrast, for another squamous cell carcinoma Tca8113 cells, no enhanced virus replication was observed (Figure 2C,D). Pretreatment with panobinostat significantly decreased the cell viability in oHSV-infected SCC9 cells in a dose-dependent manner (Figure 2E), whereas the cell viability only showed a tendency to decrease when pretreated with panobinostat at 1 nM, and the same reduction is kept even with high concentration of panobinostat at 10 or 100 nM, under either condition, with or without oHSV infection in Tca8113 cells (Figure 2F). Collectively, this suggests that pretreatment with the HDACi panobinostat facilitates oHSV replication and augments virus oncolytic activity in human glioma A172 and squamous cell carcinoma SCC9 cells. Meanwhile, cytotoxicity was also found in human glioma D54 and squamous cell carcinoma Tca8113 cells without increased oHSV replication when pretreated with panobinostat, which indicates that the enhancement of oHSV replication with panobinostat may be not associated with tumor type but cell type-dependent.

### 3.2. Treatment with Panobinostat Induces Transcriptional Downregulation of IFN-β and IFN-Stimulated Antiviral Genes in Human Glioma and Squamous Cell Carcinoma Cells

**Figure 3:**
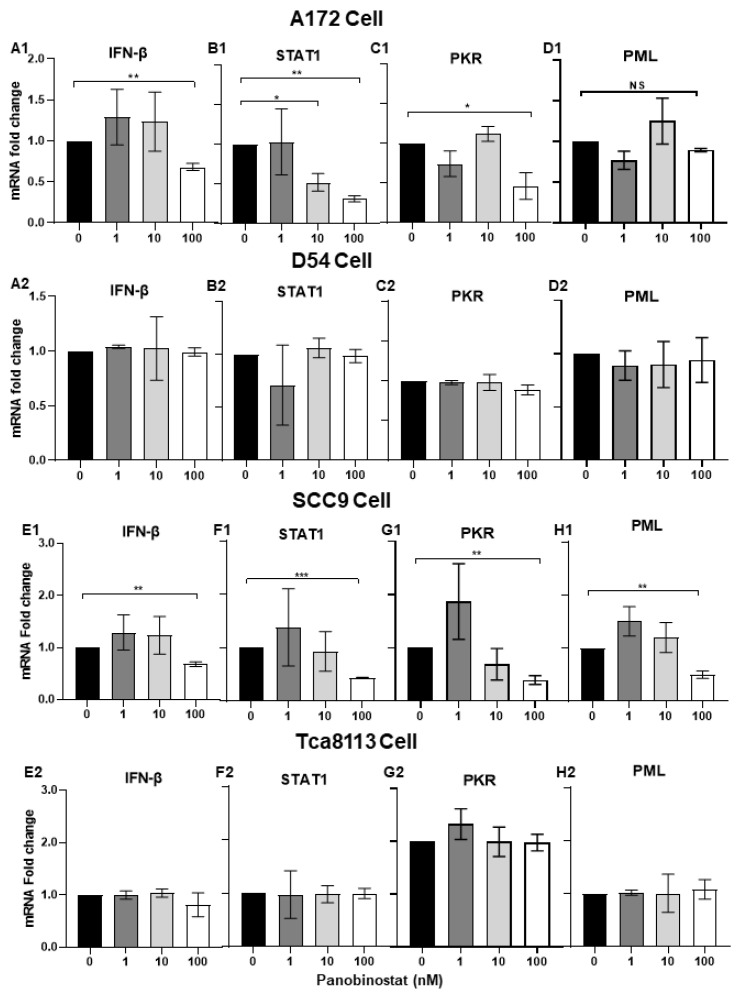
Downregulation of IFN-β- and IFN-stimulated antiviral genes when treated with panobinostat in human glioma and squamous cell carcinoma cells. A172 (**A1**,**B1**,**C1**,**D1**), D54 (**A2**,**B2**,**C2**,**D2**), SCC9 (**E1**,**F1**,**G1**,**H1**), or Tca8113 (**E2**,**F2**,**G2**,**H2**) cells were mock-treated or treated with panobinostat at different concentrations of 1, 10, or 100 nM for 14 h, respectively. Then, the cell pellets were harvested and total RNAs were extracted and reverse-transcribed to cDNA as described in the Materials and Methods section. The IFN-β, STAT1, PKR, and PML mRNAs were quantified and normalized with respect to 18S rRNA and are shown as fold changes compared to the mRNAs from the mock-treated and infected cells. Differences between datasets were assessed by Student’s *t*-test (two-tailed) using GraphPad Prism software. ^NS^ *p* >0.05, * *p* < 0.05, ** *p* < 0.01, *** *p* < 0.001.

To further investigate the mechanism behind how the combination of HDACi and oHSV enhances virus replication, the expression of IFN-β- and IFN-induced signal transducers and activators of transcription 1 (STAT1), as well as IFN-stimulated genes (ISGs) protein kinase-R (PKR), and promyelocytic leukemia (PML), were evaluated in both glioma and squamous cell carcinoma cells. It has been reported that PKR, as a host antiviral kinase, can limit γ34.5-deleted oHSV late-gene expression and replication [25] and IFN/STAT1 signaling modulators, and PML can also contribute to promoting the amplification of the antiviral response [26]. After A172, D54, SCC9, and Tca8113 cells were treated with panobinostat or mock for 14 h, and cell pellets were harvested and analyzed by RT-PCR. As shown in Figure 3(A1,B1,C1,D1), the transcriptional levels of IFN-β, STAT1, and PKR decreased significantly in A172 cells with panobinostat treatment, while PML mRNA only showed slight decrease, without significant difference. In SCC9 cells, the mRNA expression of IFN-β, STAT1, and PKR as well as PML showed a decrease with significant difference only when treated with panobinostat at 100 nM (Figure 3(E1,F1,G1,H1)). For D54 and Tca8113 cells, compared to the mock, no significant differences were shown for the expression of IFN-β, STAT1, PKR, and PML (Figure 3(A2,B2,C2,D2,E2,F2,G2,H2)). Taken together, the downregulation of IFN-β, STAT1, and PKR with panobinostat treatment indicates an inhibited antiviral state induced by HDACi, which contributes to helping oHSV escape antiviral innate immune responses and further enhances oHSV propagation in A172 and SCC9 cells.

### 3.3. Panobinostat Treatment Suppressed the Transcriptional Expression of the cGAS/STING Antiviral Innate Immune Response in Human Glioma and Squamous Cell Carcinoma Cells

**Figure 4:**
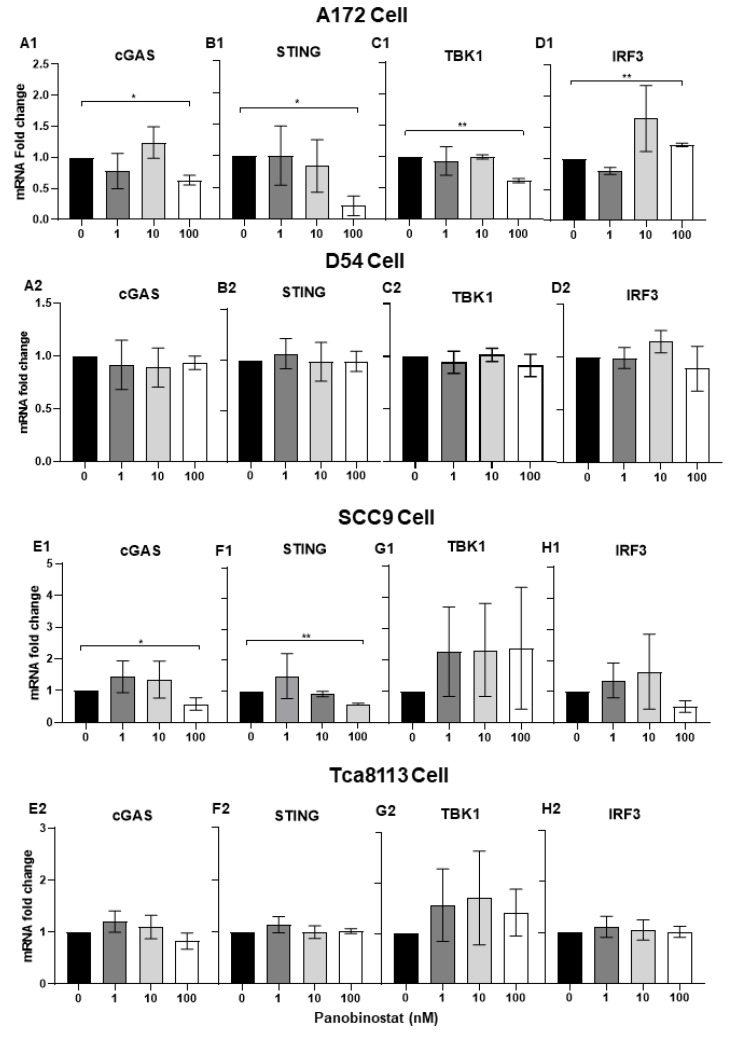
Suppression of the transcriptional expression of cGAS/STING in human glioma and squamous cell carcinoma cells when treated with panobinostat. A172 (**A1**,**B1**,**C1**,**D1**), D54 (**A2**,**B2**,**C2**,**D2**), SCC9 (**E1**,**F1**,**G1**,**H1**), or Tca8113 (**E2**,**F2**,**G2**,**H2**) cells were mock-treated or treated with panobinostat at different concentrations of 1, 10, or 100 nM for 14 h, respectively. Then, the cell pellets were harvested and total RNAs were extracted and reverse-transcribed to cDNA as described in the Materials and Methods section. cGAS, STING, TBK1, and IRF3 were quantified and normalized with respect to 18S rRNA and shown as fold changes. Differences between datasets were assessed by Student’s *t*-test (two-tailed) using GraphPad Prism software. * *p* < 0.05, ** *p* < 0.01.

It has been reported that HSV-1 can induce Interferon Type I (IFN-I) in a cGAS/stimulator of interferon gene (STING)-dependent pathway [26]. In the above study, suppressed transcriptional activation of IFN-β- and IFN-stimulated antiviral genes STAT1, PKR, and PML was observed in A172 and SCC9 cells when treated with panobinostat. Here, we further confirmed the mRNA expression of the cGAS/STING pathway, including the downstream factors TANK-binding kinase 1 (TBK1), as well as IFN regulatory factor 3 (IRF3), in A172 and SCC9 cells with panobinostat treatment. This finding shows that significant downregulation of cGAS, STING, and TBK1 were detected in A172 cells when treated with panobinostat at 100 nM, whereas an upregulation of IRF3 with the same treatment was observed (Figure 4(A1,B2,C1,D1)). In SCC9 cells, the mRNA expression of cGAS, and STING decreased significantly when treated with panobinostat at 100 nM, whereas TBK1 and IRF3 expression showed no significant difference compared to the mock treatment (Figure 4(E1,F1,G1,H1)). For D54 and Tca8113 cells, no remarkable increase or decrease for any of the cGAS/STING pathway genes detected was shown (Figure 4(A2,B2,C2,D2,E2,F2,G2,H2)). Collectively, the downregulation of cGAS and STING may act as an inhibitor of IFN-β- and IFN-stimulated antiviral genes, thus facilitating the replication of oHSV.

### 3.4. Panobinostat Treatment Induced the Upregulation of PD-L1 Expression in Glioma and Squamous Cell Carcinoma Cells

**Figure 5:**
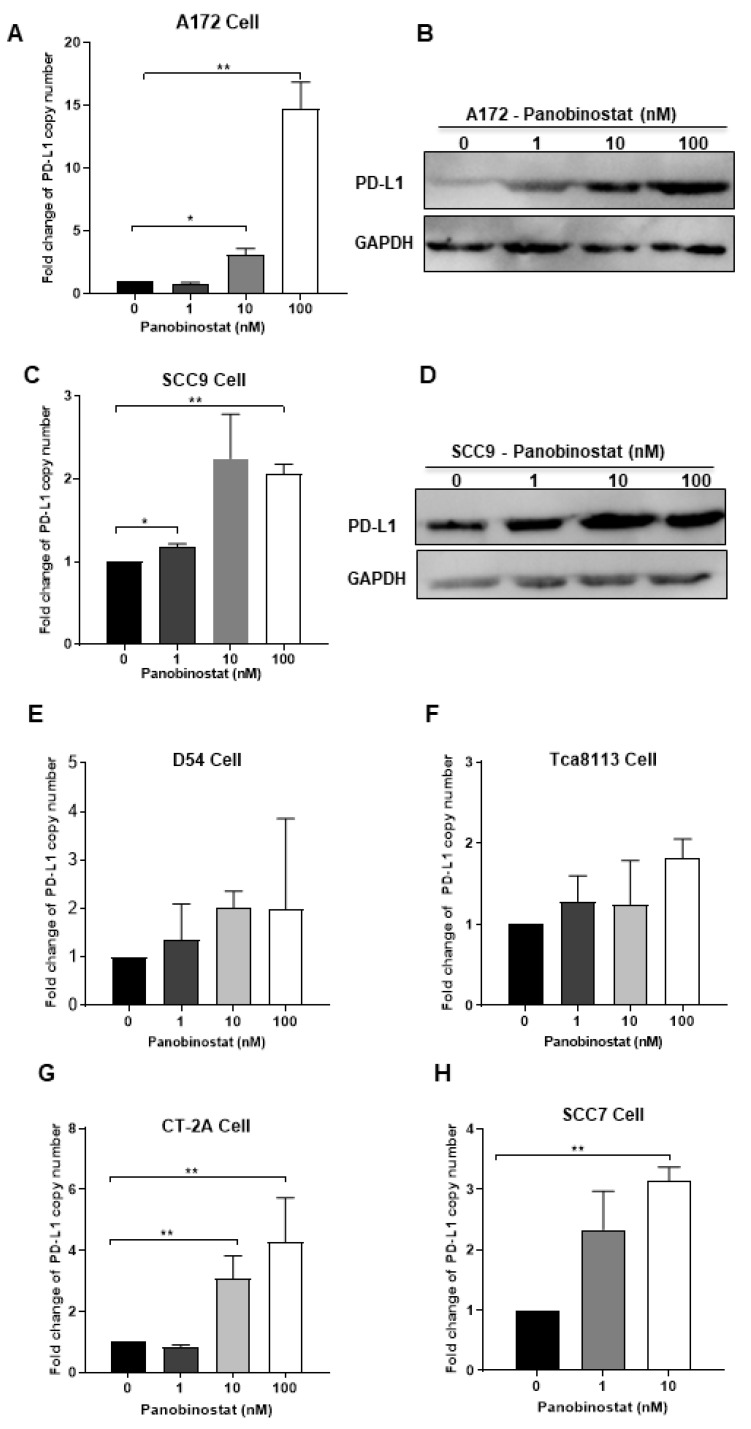
Panobinostat treatment upregulated the PD-L1 expression in glioma and squamous cell carcinoma cells. A172 (**A**,**B**), SCC9 (**C**,**D**), D54 (**E**), Tca8113 (**F**), CT-2A (**G**), or SCC7 (**H**) cells were mock-treated or treated with panobinostat at different concentrations of 1, 10, or 100 nM for 14 h, respectively. Then, the cell pellets were harvested. For each condition, half of the cell pellets were used for total RNA extraction, following reverse transcription to cDNA. The other half of the cell pellets were lysed and used for Western blot (WB) as described in the Materials and Methods section. The assay was performed in triplicate. Differences between datasets were assessed by Student’s *t*-test (two-tailed) using GraphPad Prism software. * *p* < 0.05, ** *p* < 0.01.

It has been reported that high expression of PD-L1 in tumors is one of the biomarkers for improved sensitivity to PD-1/PD-L1 blockade [27]. In the next in vivo study, the oHSV T3855 [24], with the same genomic backbone as T1012G and containing murine IL-12 and PD-1 antibody, was used. To confirm the possible effects of panobinostat treatment on PD-L1 expression, the transcriptional and translational levels of PD-L1 were detected in A172 and SCC9 cells following panobinostat treatment. As shown in Figure 5A, the mRNA expression of PD-L1 increased significantly (*p* < 0.01), around 15-fold for A172 cells at a concentration of 100 nM. Moreover, the PD-L1 protein expression showed a similar increasing pattern to the mRNAs, in a dose-dependent manner (Figure 5B). For another glioma cell D54, treatment with panobinostat showed no significant difference on PD-L1 mRNA level (Figure 5E). For SCC9 cells treated with panobinostat at 100 nM, an around-two-fold increase was observed on the PD-L1 mRNA level, with a significant difference compared to the mock treatment (*p* < 0.01) (Figure 5C). Meanwhile, the PD-L1 protein expression slightly increased when treated with panobinostat (Figure 5D). For Tca8113, another human squamous cell carcinoma cell, the mRNA level of PD-L1 only displayed a slight increase when treated with panobinostat at 100 nM (Figure 5F). For both murine glioma CT-2A cells and murine squamous cell carcinoma SCC7 cells, the transcriptional expression of PD-L1 was significantly increased when treated with panobinostat (Figure 5G,H). As a high concentration of panobinostat at 100 nM imparts serious cytotoxicity to SCC7 cells, the maximum concentration used for SCC7 was 10 nM. Taken together, the upregulation of PD-L1 was shown in glioma and squamous cell carcinoma cells following panobinostat treatment, which may contribute to PD-1/PD-L1 blockade.

### 3.5. Combined Treatment with HDACi Panobinostat and oHSV T3855 Further Enhanced the Antitumor Therapeutic Activity in Murine Glioma and Squamous Cell Carcinoma Models

The in vitro study above showed that HDACi panobinostat treatment can enhance oHSV replication and augment virus oncolytic activity, as well as upregulate PD-L1 expression in glioma and squamous cell carcinoma cells. Thus, whether combination therapy with panobinostat and oHSV T3855 can enhance the antitumor therapeutic efficacy in vivo was investigated. Syngeneic tumor mice were implanted with murine glioma model CT-2A or murine squamous cell carcinoma SCC7, respectively.

As cytotoxicity of SCC7 cells was shown when treated with high dosing of HDACi panobinostat, the proper concentration of panobinostat was also selected for the in vivo study. Given treatment with panobinostat only, at a concentration of 5 mg/kg or 20 mg/kg, no animals were dead at the end of the study (Day 15), though 20 mg/kg dosing induced around 15% weight loss of mice. When combined with oHSV T3855, a 75% death rate on day 10 was shown in the high-concentration group (20 mg/kg) (Table S1). Thus, 5 mg/kg of panobinostat was used for the following study.

**Figure 6:**
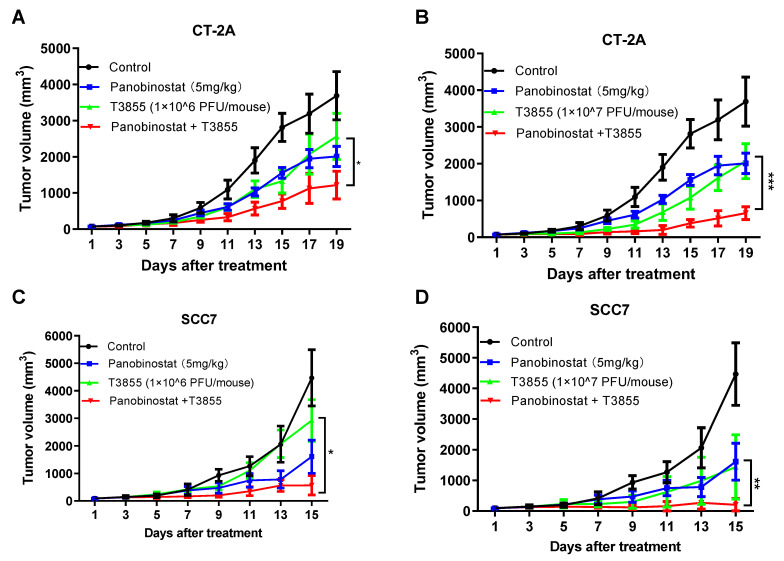
HDACi panobinostat and oHSV T3855 combination therapy further enhanced the antitumor efficacy in the murine glioma and squamous cell carcinoma models. CT-2A (**A**,**B**) or SCC7 (**C**,**D**) tumor cells were injected s.c. into the right flanks of syngeneic mice, respectively. Different tumor models averaging 80 mm^3^ (*n* = 8 per group) were treated via intratumoral injection with PBS or T3855 (1 × 10^6^ or 1 × 10^7^ PFU/animal) on Day 1, and HDACi (panobinostat; 5 mg/kg) or the vehicle was given via intraperitoneal injection with a five-day-on and two-day-off regimen for two weeks. The tumor volumes are shown as the mean ± SEM of eight animals in each group. Differences between datasets were assessed by Student’s *t*-test (two-tailed) using GraphPad Prism software. * *p* < 0.05, ** *p* < 0.01, *** *p* < 0.001.

As shown in Figure 6A,B, delayed tumor growth of CT-2A was observed with panobinostat alone and oHSV T3855 alone, while combined treatment was associated with a significant decrease in tumor volume compared to oHSV T3855 alone at 1 × 10^6^ GPFU/mouse (*p* < 0.05) or 1 × 10^7^ PFU/mouse (*p* < 0.001). For murine squamous cell carcinoma SCC7, similarly to CT-2A, combined treatment showed an obvious decrease in tumor growth compared to oHSV alone at 1 × 10^6^ PFU/mouse (*p* < 0.05) or 1 × 10^7^ PFU/mouse (*p* < 0.01) (Figure 6C,D). Furthermore, during combination therapy with 1 × 10^7^ PFU/mouse of oHSV, three of the eight mice showed a complete response in the SCC7 model after 15 days’ treatment (Table S2). This suggests that in both the CT-2A and SCC7 models, a higher concentration of oHSV displayed enhanced antitumor therapeutic activity compared to a lower concentration of oHSV, and combined panobinostat treatment with oHSV further amplified the anti-tumor therapeutic effect.

## 4. Discussion

To improve the oncolytic activity of oncolytic virus monotherapy, the HDACi panobinostat was used in combination with oHSV in this study, and we provide evidence that pretreatment with panobinostat enhances oHSV replication in vitro. Moreover, augmented therapeutic efficacy was shown in vivo for the glioma and squamous cell carcinoma models. Enhancement of oncolytic therapy, or overcoming the resistance of tumor cells to oncolytic virus, includes increasing viral susceptibility to tumor cells and decreasing the inhibition effect on virus infection from innate antiviral immune responses, as well as facilitating apoptotic tumor death [4,5,6]. Some host factors mentioned in virus entry and egress are also important to increase the viral susceptibility to tumor cells [28]. It has been reported that a class IIa HDACi (LMK-235) upregulates the receptor of oHSV nectin-1 on human hematological Ramos and HEL cells without substantially reducing cell viability, and further significantly enhances viral entry in a dose-dependent manner [29]. However, this is inconsistent with our results, as there was no significant change in nectin-1 expression when treated with panobinostat in glioma A172 and squamous cell carcinoma SCC9 cells (Figure S4). This may be related to the various expression basal lines of nectin-1 in different tumor cells.

Several previous studies have also described the suppression of innate antiviral immune responses induced by HDAC inhibition when combined with oncolytic viruses. Candace et al. reported that HDACi can enhance cell killing and block the IFN-β synthesis elicited by infection with an oncolytic parainfluenza virus in human-airway cancer cells [30]. The pre-addition of HDACi with conditionally replicating adenovirus injection has shown synergistic antitumor effects [31]. For oHSV, VPA pre-administration enhances oHSV activity by inducing a decline in NK and macrophage recruitment into tumor-bearing brains at 6 and 24 hpi [32]. In addition, pretreatment with VPA also inhibits the induction of several IFN-responsive antiviral genes, augments the transcriptional level of oHSV genes, and improves viral propagation [16]. In another breast cancer model, pre-treatment with HDAC inhibitors also showed enhanced replication of oHSV [33]. These coincide with our findings that pretreatment, but not co-treatment, with HDACi panobinostat enhances oHSV replication, which may be mediated by downregulation of the transcriptional levels of IFN-β- and IFN-responsive antiviral genes in glioma A172 and squamous cell carcinoma SCC9 cells. Actually, in addition to 14 h pretreatment, the time points 24 h and 36 h were also performed, but no more enhancement of virus replication could be observed, and more cytotoxicity was shown along the time. Thus, only 14 h pretreatment was used in this study. Taken together, this suggests that in oncolytic therapy, HDACi could create a favorable immune environment for therapeutic viruses. What is more, the timing of HDACi administration is critical for the effectiveness of combined treatment with optimal OV therapy.

The cGAS/STING-mediated DNA-sensing signaling pathway is crucial for IFN production and host antiviral responses, and HSV-1 can induce IFN-I in a cGAS/STING-dependent pathway [34,35]. cGAS has been identified as the principal sensor recognizing cytosolic aberrant DNA to produce cGAMP via its enzymatic activity [35]. Then, cGAMP acts as an important secondary messenger to activate STING and recruits TBK1 to activate IRF3. Next, IRF3, the pivotal transcription factor of IFN-β, binds to the promoter of IFN-β to boost the expression of IFN-β [36,37,38]. Here, we observed the transcriptional downregulation of IFN-β and GAS/STING in A172 and SCC9 cells with panobinostat treatment. However, the upregulation of downstream IRF3 was shown in A172 cells. It has been reported that a member of class II, HDAC4, regulates antiviral response by inhibiting the phosphorylation of IRF3 [39] and panobinostat is a pan-deacetylase inhibitor with activity against class I, II, and IV HDAC. Together, this indicates that suppression of the cGAS/STING pathway may contribute to the inhibition of IFN-β, as well as IFN-inducible genes, further facilitating the replication of oHSV.

HDACi can induce cell-cycle arrest and apoptosis in transformed cells, including tumor cells [40]. Additionally, our studies verified cytotoxicity with panobinostat alone, and significantly increased cell killing combined with oHSV in human glioma and squamous cell carcinoma cells. In addition to cytotoxicity, a wide range of immunologic changes in tumor cells can also be induced by HDACi, including enhanced MHC class I and II surface expression and increased expression of differentiation antigens, as well as other immunologically relevant costimulatory molecules [41,42,43]. Several studies have shown that HDACi can reduce “negative” cell populations, such as myeloid-derived suppressor cells, and can augment checkpoint blockade therapies, such as PD-1/PD-L1 blockade [44,45,46]. Here, within human and murine glioma and squamous cell carcinoma cells, remarkable upregulation of PD-L1 was observed, and PD-1/PD-L1 blockade was verified via a competition ELISA (unpublished data), which further confirms the connection between epigenetics and immune regulation and the promising combination therapy with PD-1/PD-L1 blockade and HDACi.

In the in vivo study, therapeutic effectiveness was seen in the syngeneic tumor mice. The combination of oHSV T3855 with HDACi contains the oncolytic effects of oHSV, PD-1 blockade, and HDAC inhibition. The oHSV T3855 was used in vivo expressing murine IL-12 and murine scFV (single-chain variable fragment) antibody against PD-1. We found that, in both the murine glioma and squamous cell carcinoma models, the combined treatment of oHSV T3855 with HDACi showed significant inhibition of tumor progression, which may be due to increased virus replication (Figure S3) and upregulated PD-L1 expression, as we determined in vitro. Moreover, PD-L1 upregulation has been indicated to convert a cold tumor into a hot tumor. In addition, it has been reported that HDACi treatment can contribute to anti-angiogenesis in glioma, which is crucial for anti-tumor response [45]. In the squamous cell carcinoma SCC7 model, combination therapy with 10^7^ PFU/mouse of oHSV administration showed 37.5% complete tumor eradication, while there was no complete response shown in the CT-2A model. In vitro, SCC7 also showed a relatively higher sensitivity to panobinostat. This may be relevant to the different tumor progressions and cell type-dependent sensitivities to panobinostat. In this study, only a subcutaneous tumor model was studied, considering that HDACi panobinostat can penetrate the BBB and achieve effective brain concentration in a murine model [46]. Thus, orthotopic mouse brain tumor models will be further explored in the future.

In summary, combined treatment with oHSV and HDACi panobinostat augmented virus replication, mediated by the downregulation of IFN-β- and IFN-stimulated antiviral genes, as well as the cGAS/STING pathway in glioma and squamous cell carcinoma cells. In vivo, enhanced therapeutic efficacy was shown in combination with oHSV expressing the PD-1 blockade antibody and HDAC inhibition in the glioma and squamous cell carcinoma models. Notably, only pretreatment and not co-treatment showed enhanced oHSV replication; thus, the administration timing and length of treatments are also critical factors that need to be considered when utilizing a combination therapy with HDAC inhibitors and oncolytic viruses. Collectively, these data provide some new clues for the clinical development of combination therapy with OVs, epigenetic modifiers, and checkpoint blockades for glioma and squamous cell carcinoma.

## 5. Conclusions

Treatment with the HDACi panobinostat augmented the oHSV oncolytic activity in glioma and squamous cell carcinoma cells. Combined treatment with oHSV and the HDACi panobinostat enhanced virus replication mediated by the downregulation of IFN-β- and IFN-stimulated antiviral genes, as well as the cGAS/STING pathway in glioma and squamous cell carcinoma cells. An enhanced therapeutic efficacy was shown in combination with oHSV expressing the PD-1 blockade antibody and HDAC inhibition in the glioma and squamous cell carcinoma models.

## Figures and Tables

**Table 1:** Primers used for amplifications.

Gene	Forward Primer	Reverse Primer
Human *IFN-β*	5′-ATGACCAACAAGTGTCTCCTCC-3′	5′-GGAATCCAAGCAAGTTGTAGCTC-3′
Human *STAT1*	5′-CAGCTTGACTCAAAATTCCTGGA-3′	5′-TGAAGATTACGCTTGCTTTTCCT-3′
Human *PKR*	5′-GCCGCTAAACTTGCATATCTTCA-3′	5′-TCACACGTAGTAGCAAAAGAACC-3′
Human *PML*	5′-CGCCCTGGATAACGTCTTTTT-3′	5′-CTCGCACTCAAAGCACCAGA-3′
Human *cGAS*	5′-ACCCAGAACCCTCAAGAC-3′	5′-GAGGCACTGAAGAAAGTATGTC-3′
Human *STING*	5′-CCTGAGTCTCAGAACAACTGCC-3′	5′-GGTCTTCAAGCTGCCCACAGTA-3′
Human *TBK1*	5′-TGGGTGGAATGAATCATCTACGA-3′	5′-GCTGCACCAAAATCTGTGAGT-3′
Human *IRF3*	5′-GCCGAGGCCACTGGTGCATAT-3′	5′-TGGGTCGTGAGGGTCCTTGCT-3′
Human *PD-L1*	5′-CAAAGAATTTTGGTTGTGGA-3′	5′-AGCTTCTCCTCTCTCTTGGA-3′
Mouse *PD-L1*	5′-AGCTGAATTGGTCATCCCAGAA-3′	5′-CAATGGACTGTGTCAGTGTTGT-3′
Human *18S*	5′-CTCAACACGGGAAACCTCAC-3′	5′-CGCTCCACCAACTAAGAACG-3′

## Supplementary Materials

The following supporting information can be downloaded at: https://www.mdpi.com/article/10.3390/v14122796/s1. Figure S1: Differential capacity of oHSV-1 T1012G replicating in human glioma cell and squamous cell carcinoma cell lines (A) and panobinostat treatment induces histone acetylation for both histones H3 and H4 in human glioma cell and squamous cell carcinoma cell lines (B). Figure S2: Co-treatment with panobinostat and oHSV showed almost no increase in the viral titer in human glioma A172 cells (A,B) and D54 cells (C,D). Figure S3: The HDACi panobinostat promoted oHSV replication in murine glioma CT-2A cells (A,B) and squamous cell carcinoma SCC7 cells (C,D). Figure S4: The expression of nectin-1 when treated with panobinostat in glioma A172 cells (A) and squamous cell carcinoma SCC9 cells (B). Table S1: The survival rate was summarized when combination therapy of oHSV T3855 and HDACi panobinostat at different concentrations was performed in squamous cell carcinoma SCC7 model. Table S2: Complete tumor eradication (CR) was summarized when HDACi panobinostat and oHSV T3855 combination therapy were performed in the murine glioma CT-2A and squamous cell carcinoma SCC7 models.
