# Supplementary material for: Histone Deacetylase Inhibitor Panobinostat Benefits the Therapeutic Efficacy of Oncolytic Herpes Simplex Virus Combined with PD-1/PD-L1 Blocking in Glioma and Squamous Cell Carcinoma Models
Source: Viruses. 2022 Dec 15;14(12):2796. doi: 10.3390/v14122796 (PMC9781547; doi:10.3390/v14122796)
Supplement: Supplementary file 1 [file viruses-14-02796-s001.zip › viruses-2057205-supplementary.pdf]

viruses

MDPI

## Supplementary Materials

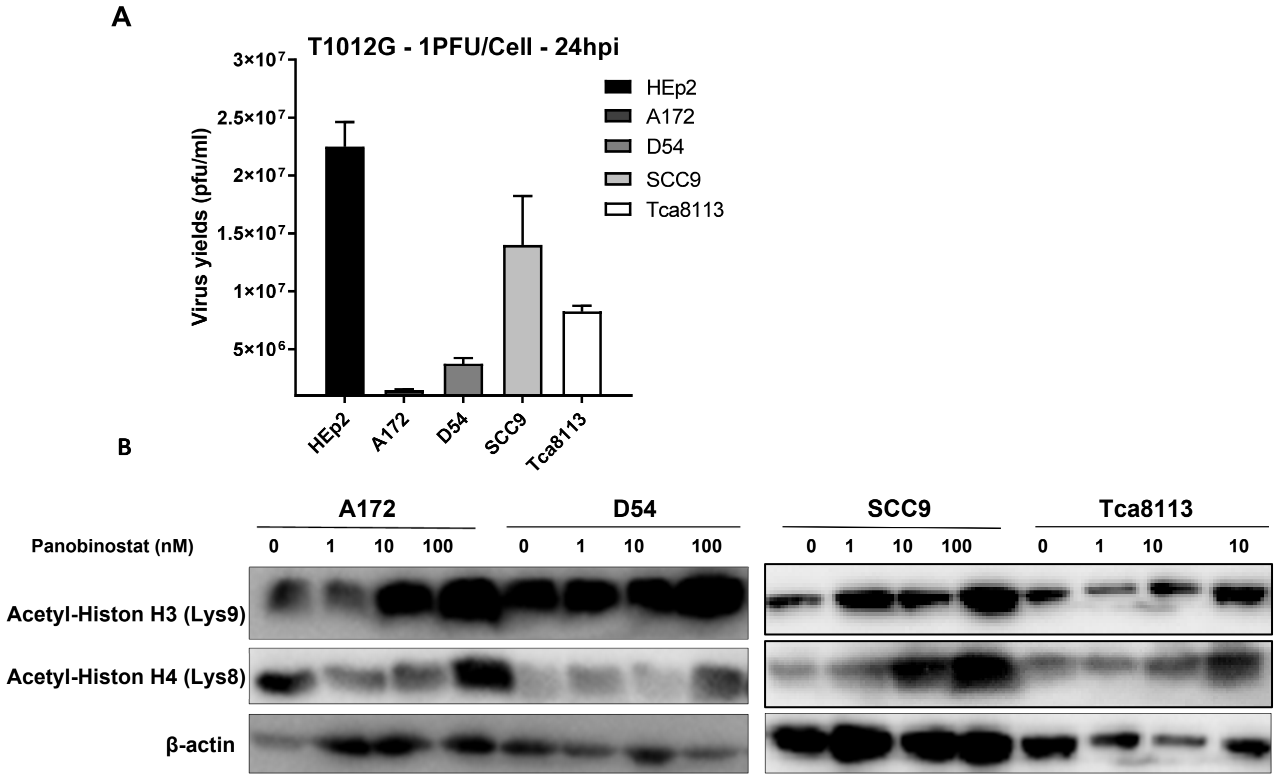


**Figure S1.** Differential capacity of oHSV-1 T1012G replicating in human glioma cell and squamous cell carcinoma cell lines (**A**) and panobinostat treatment induces histone acetylation for both histones H3 and H4 in human glioma cell and squamous cell carcinoma cell lines (**B**)

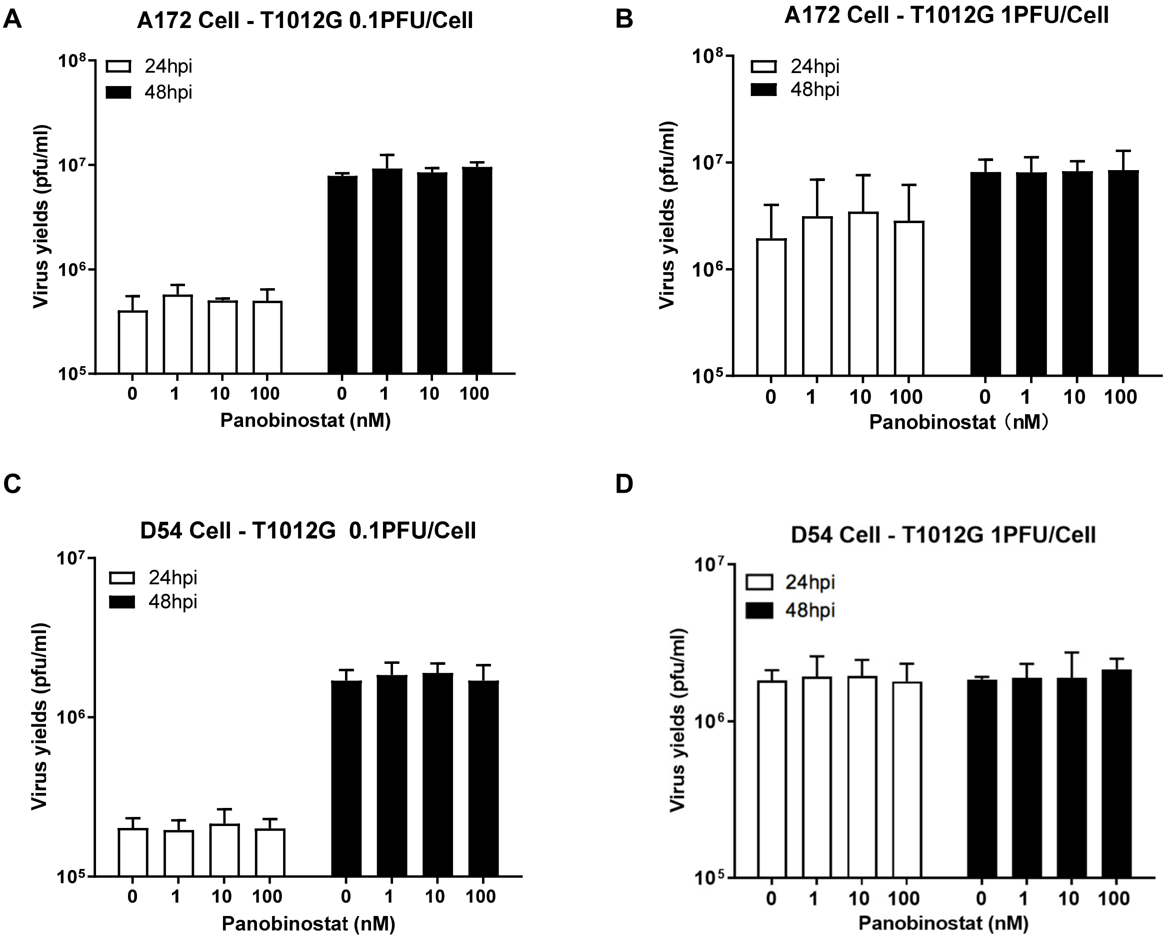


**Figure S2.** Co-treatment with panobinostat and oHSV showed almost no increase in the viral titer in human glioma A172 cells (**A,B**) and D54 cells (**C,D**). A172 (A, B) and D54 (C, D) were treated with panobinostat at concentrations of 1, 10, and 100 nM and meanwhile, infected with T1012G (0.1 or 1 PFU/Cell) for 48 h, respectively. The infected cell pellets were harvested at 48 hpi. The titration was measured by conventional plaque assay on Vero cells after three freeze–thaw cycles. The assay was performed in triplicate. Differences between datasets were assessed by student's t-test (two-tailed) using the GraphPad Prism software.

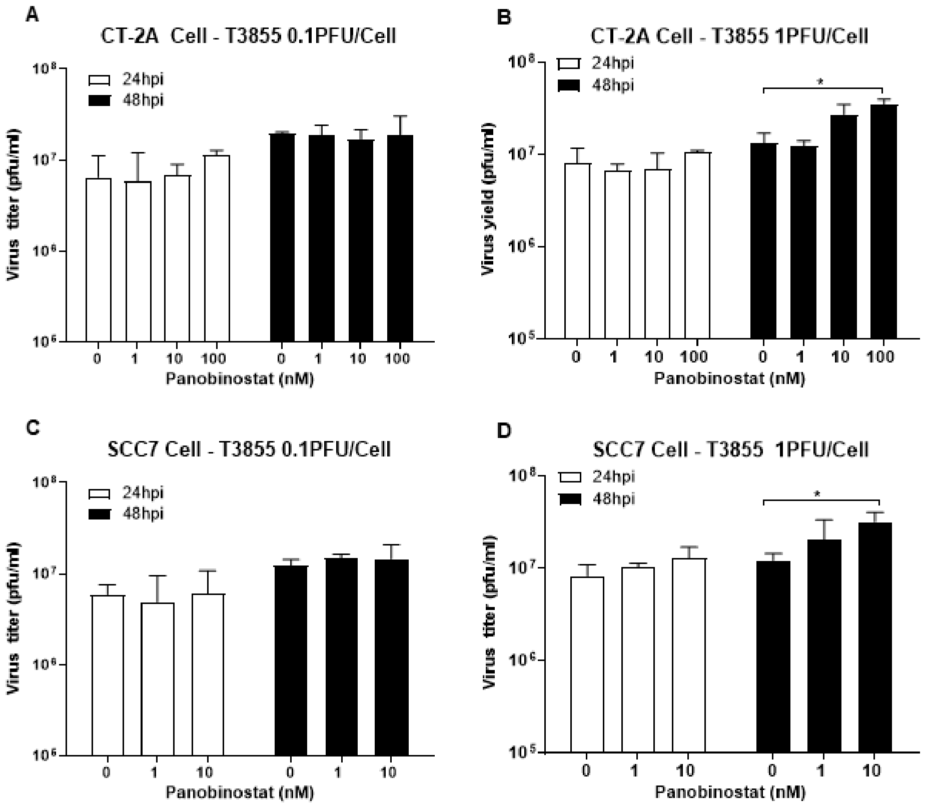


**Figure S3.** The HDACi panobinostat promoted oHSV replication in murine glioma CT-2A cells (**A,B**) and squamous cell carcinoma SCC7 cells (**C,D**). CT-2A (**A, B**) cells were pretreated with panobinostat at concentrations of 1, 10, and 100 nM for 14 h and then infected with T3855 (0.1 or 1 PFU/cell) for another 48 h, respectively. SCC7 (**C, D**) were pretreated with panobinostat at concentrations of 1 and 10 nM for 14 h and then infected with T3855 (0.1 or 1 PFU/cell) for another 48 h, respectively. The infected cell pellets were harvested at 48 hpi. The titration was measured by conventional plaque assay on Vero cells after three freeze–thaw cycles. The assay was performed in triplicate. Differences between datasets were assessed by student's t-test (two-tailed) using the GraphPad Prism software. * $p < 0.05$.

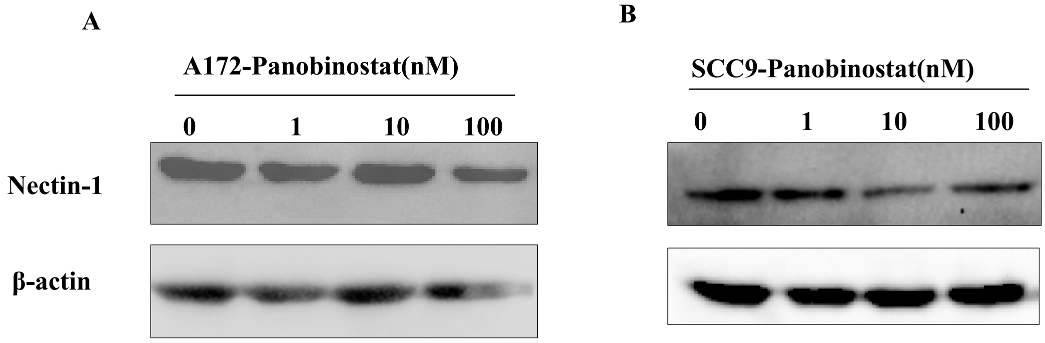


**Figure S4.** The expression of nectin-1 when treated with panobinostat in glioma A172 cells (**A**) and squamous cell carcinoma SCC9 cells (**B**). A172 (**A**) or SCC9 (**B**) cells were mock-treated or treated with panobinostat at different concentrations of 1, 10, or 100 nM for 14 h, respectively. Then, the cell pellets were harvested, lysed and used for Westen blot (WB), as described in the Materials and Methods section.

**Table S1.** The survival rate was summarized when combination therapy of oHSV T3855 and HDACi panobinostat was performed in squamous cell carcinoma SCC7 model. The concentrations of HDACi panobinostat used include 5 mg/kg and 20 mg/kg.

| **Groups (N=8)** | **No. of survival mice/ No. of all mice** | | | | | | | |
|---|---|---|---|---|---|---|---|---|
| SCC7 | **D1** | **D3** | **D5** | **D7** | **D9** | **D11** | **D13** | **D15** |
| **Control** | **8/8** | **8/8** | **8/8** | **8/8** | **8/8** | **8/8** | **8/8** | **8/8** |
| **Panobinostat (5 mg/kg)** | **8/8** | **8/8** | **8/8** | **8/8** | **8/8** | **8/8** | **8/8** | **8/8** |
| **Panobinostat (20 mg/kg)** | **8/8** | **8/8** | **8/8** | **8/8** | **8/8** | **8/8** | **8/8** | **8/8** |
| **T3855 (1 × $10^6$ PFU/mouse)** | **8/8** | **8/8** | **8/8** | **8/8** | **8/8** | **8/8** | **8/8** | **6/8** |
| **T3855 (1 × $10^7$ PFU/mouse)** | **8/8** | **8/8** | **8/8** | **8/8** | **8/8** | **8/8** | **8/8** | **7/8** |
| **Panobinostat（5 mg/kg）+ T3855 (1 × $10^6$ PFU/mouse)** | **8/8** | **8/8** | **8/8** | **8/8** | **8/8** | **8/8** | **8/8** | **8/8** |
| **Panobinostat（5 mg/kg）+ T3855 (1 × $10^7$ PFU/mouse)** | **8/8** | **8/8** | **8/8** | **8/8** | **8/8** | **7/8** | **7/8** | **7/8** |
| **Panobinostat（20 mg/kg）+ T3855 (1 × $10^6$ PFU/mouse)** | **8/8** | **8/8** | **7/8** | **7/8** | **7/8** | **3/8** | **4/8** | **2/8** |
| **Panobinostat（20 mg/kg）+ T3855 (1 × $10^7$ PFU/mouse)** | **8/8** | **8/8** | **8/8** | **7/8** | **6/8** | **0/8** | **0/8** | **0/8** |

**Table S2.** Complete tumor eradication (CR) was summarized when HDACi panobinostat and oHSV T3855 combination therapy were performed in the murine glioma CT-2A and squamous cell carcinoma SCC7 models.

| Index<br>Groups<br>(N=8) | No. of CR mice/ No. of survival mice | | | |
|---|---|---|---|---|
| | CT-2A | | SCC7 | |
| | (1 x $10^6$ PFU/mouse ) | (1 x $10^7$ PFU/mouse ) | (1 x $10^6$ PFU/mouse ) | (1 x $10^7$ PFU/mouse ) |
| **Control** | **0/4** | **0/4** | **0/4** | **0/4** |
| **T3855** | **0/6** | **0/7** | **0/6** | **0/7** |
| **Panobinostat: 5 mg/kg** | **0/8** | **0/8** | **0/8** | **0/8** |
| **T3855 + Panobinostat** | **0/8** | **0/8** | **0/8** | **3/8** |
